# Surveillance of Healthcare-Associated Infections in Long-Term Care Facilities in Graz, Austria, from 2018 to 2022

**DOI:** 10.3390/antibiotics14060573

**Published:** 2025-06-03

**Authors:** Elisabeth König, Miriam Meister, Christian Pux, Michael Uhlmann, Walter Schippinger, Herwig Friedl, Robert Krause, Ines Zollner-Schwetz

**Affiliations:** 1 Division of Infectious Diseases, Department of Internal Medicine, Medical University of Graz, 8036 Graz, Austria; 2 Geriatric Health Centers of the City of Graz, 8020 Graz, Austria; christian.pux@stadt.graz.at (C.P.);; 3 Institute of Statistics, Graz University of Technology, 8010 Graz, Austria; hfriedl@tugraz.at

**Keywords:** nursing home, surveillance, elderly, nosocomial infections

## Abstract

**Objectives:** This study aimed to evaluate changes in the rate and spectrum of healthcare-associated infections (HCAIs) and to analyse the rate and spectrum of antimicrobial prescriptions in four long-term care facilities (LTCFs) in Graz, Austria, from 2018 to 2022 in a prospective cohort study. **Methods:** Nursing staff prospectively collected data on HCAIs and antimicrobial prescriptions once a week. Log-linear Poisson models for counts were applied mostly to evaluate the difference effects of the various calendar years compared to the reference year of 2018. **Results:** A total of 1684 infections were recorded in 720 residents during the study period. The overall annual incidence rate of HCAIs varied over time with a significant increase to 2.86/1000 resident days in 2019 and to 4.09/1000 resident days in 2022, both compared to 2018, *p* < 0.001. A large peak in respiratory tract infections (RTIs) occurred in winter 2021/2022 due to a large number of SARS-CoV-2 infections in all four LTCFs. Urinary tract infections (UTIs) were the most commonly recorded infections. Beta-lactams were the most frequently prescribed systemic anti-infectives. A statistically significant increase in the rate of beta-lactam prescriptions/1000 resident days occurred between 2018 and 2022 (*p* = 0.016), whereas a statistically significant decrease in quinolone prescriptions/1000 resident days occurred in the same time period (*p* < 0.001). **Conclusions:** The incidence rates of HCAIs varied over time with a significant increase during the COVID-19 pandemic in 2022 compared to 2018. Continued surveillance efforts are necessary to assess the effect of infection control efforts after the pandemic.

## 1. Introduction

By 2050, the European Commission predicts that the proportion of people in Europe over 65 will be approximately 30%, compared to about 20% today [1]. An aging population leads to many challenges for healthcare systems. Long-term care facilities (LTCFs) for the elderly already play an essential role in contemporary healthcare systems due to the demographic changes in the industrialised world. Approximately 2–5% of the population of high-income countries reside in some type of long-term care facility (LTCF) [2]. These residents are at an increased risk of nosocomial and healthcare-associated infections (HCAIs) due to age-related factors, such as immunosenescence, a decline in functional status, chronic comorbidities, the use of invasive medical devices, dependence on care and grouped living conditions [3,4,5]. Infections at LTCFs are a common cause of residents’ mortality and morbidity, and are associated with a significant socio-economic burden [6]. Among residents of Norwegian LTCFs, the acquisition of a HCAI was associated with a reduction in overall physical condition, as well as an increase in hospital admissions and mortality [7].

The prevalence of HCAIs in LTCFs varies in different settings. In 2016 and 2017, the European Centre for Disease Prevention and Control (ECDC) organised point prevalence surveys on HCAIs in acute care hospitals and LTCFs [8]. HCAI prevalences in LTCFs ranged from 0.6% in Lithuania to 6.2% in Spain, with an average of 3.1% (HCAI with origin in own LTCF) [8]. In Switzerland, the prevalence was found to be 4.2% in 2019 [9]. A Dutch annual point prevalence study found HCAI prevalences ranging from 6.7% to 7.6% in the years 2007–2009 [10].

Incidence studies may give different and more detailed insights than point prevalence studies [10]. In 2018, prospective surveillance of HCAIs was started at the LTCFs of the Geriatric Health Centres in Graz, Austria as part of the infection control and prevention program. The overall incidence rate was 2.1/1000 resident days in 2018 [11]. Urinary tract infections (UTIs) were the most commonly recorded HCAIs, followed by skin and respiratory tract infections (RTIs) [11]. In comparison, studies from German LTCFs from 1998/1999 and 2006 had documented incidence rates between 5 and 6/1000 resident days [12,13].

Surveillance of HCAIs is important to detect outbreaks, changes in infection rates and other issues requiring an infection control intervention [14]. Continued surveillance efforts have been described to contribute to a decrease in HCAIs, possibly due to an increased awareness [15,16,17]. The purpose of this study was to continuously evaluate the rate of HCAIs and antimicrobial use in four Austrian LTCFs over a period of five years. In particular, the aims of this study were to evaluate changes in the rate of HCAIs, to characterise the spectrum of HCAIs and to analyse the rate of antimicrobial prescriptions in the four LTCFs from 2018 to 2022.

## 2. Results

### 2.1. Patient Characteristics

The mean age of the residents with recorded infections was 85.7 ± 8.6 years and 74.7% (538/720) of the residents were female. There were no statistically significant differences in the mean ages of the residents with recorded infections between the years.

### 2.2. Incidence Rate of Healthcare-Associated Infections

A total of 1684 infections were recorded in 720 residents during the study period. The overall annual incidence rate of HCAIs varied over time, with a significant increase to 2.86/1000 resident days in 2019 and to 4.09/1000 resident days in 2022, both compared to 2018, *p* < 0.001 (Table 1).

The following disease entities were documented: lower respiratory tract infections (RTI), urinary tract infections (UTI), skin and mucosal infections (including infections of the eyes, ears and teeth), gastrointestinal infections (GTI) and unexplained febrile illness.

In the years 2018–2021, UTIs were the most commonly recorded infections (43–49%). In 2022, RTIs were most common (51%).

**Figure 1:**
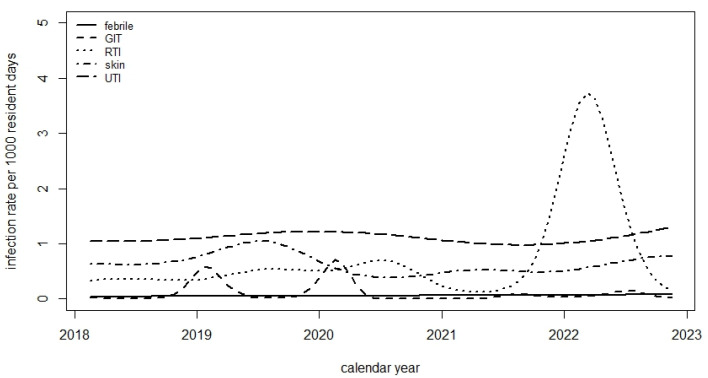
Infection rate per 1000 resident days in five disease categories. Febrile = unexplained febrile illness. GTI = gastrointestinal infection. RTI = respiratory tract infection. Skin = skin and mucosal infections (including infections of the eyes, ears and teeth). UTI = urinary tract infection.

Statistically significant changes in incidence rates of infections/1000 resident days occurred in the following categories between 2018 and 2022: respiratory tract infections (RTI), gastrointestinal tract infections (GTI) and skin infections. A large peak in RTIs occurred in winter 2021/2022 due to a large number of SARS-CoV-2 infections in all four LTCFs during this time. Two peaks in GTIs that occurred in early 2019 and early 2020 were due to norovirus outbreaks in two LTCFs. Overall, no changes over time occurred in the UTI and unexplained febrile illness categories (Figure 1). There were statistically significant differences in the mean rates of UTIs between different LTCFs (*p* < 0.001).

### 2.3. Rate of Antimicrobial Prescriptions

**Figure 2:**
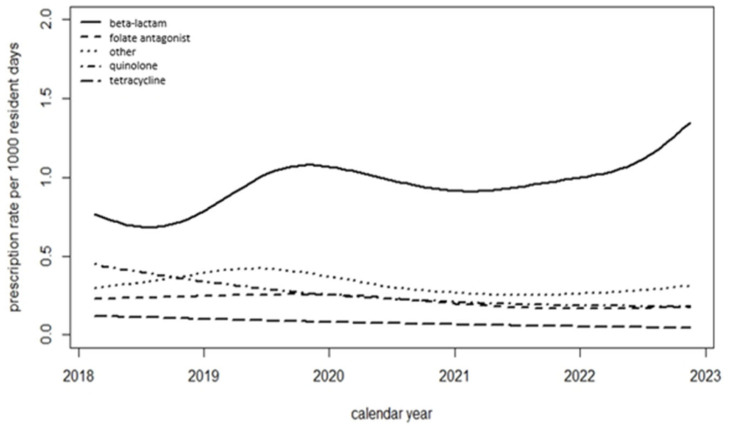
Prescription rates per 1000 resident days for 5 different anti-infective groups from 2018 to 2022.

The prescription rate/1000 resident days of systemic antimicrobial substances was significantly higher in 2019 compared to 2018 (2.13 vs. 1.77, *p* < 0.05). No significant changes occurred in the years 2020 to 2022 compared to 2018 (Table 2). Beta-lactams were the most frequently prescribed systemic anti-infectives (Figure 2). A statistically significant increase in the rate of beta-lactam prescriptions/1000 resident days occurred between 2018 and 2022 (*p* = 0.016), whereas a statistically significant decrease in quinolone prescriptions/1000 resident days occurred in the same time period (*p* < 0.001; Figure 2). No changes were found in the other three groups.

## 3. Discussion

The incidence rate of HCAIs in four LTCFs varied from 1.86/1000 resident days in 2021 to 4.08/1000 resident days in 2022. A similar study conducted in six long-term care hospitals in South Korea in 2019 reported a lower incidence rate of 1.57 per 1000 resident days [18]. In comparison, studies from German LTCFs from 1998/1999 and 2006 documented incidence rates between 5 and 6/1000 resident days [12,13]. A study from Norway reported an incidence rate of 5.2 per 1000 resident days in the years 2004 and 2005 [7]. Divergence in the definitions used, settings (such as staffing ratios, availability of onsite physicians, etc.) and timing (pre-pandemic vs. during the pandemic) could explain these different findings.

The HCAI peak in early 2022 was caused by a large number of SARS-CoV-2 infections in all four LTCFs. On March 15, 2022, the highest incidence rate of SARS-CoV-2 infections ever recorded in the general Austrian population during the pandemic was documented [19]. The Omicron variants BA1 and BA2 were predominant at this time and vaccinations against these variants were only started in September 2022 in Austria [19]. These factors could explain the peak in RTIs in our study in the beginning of 2022.

In 2019, the overall incidence rate was significantly higher compared to 2018, probably due to a norovirus outbreak in one of the facilities. In addition, an unclear rise of documented skin infections occurred in 2019. During the study period, no changes in incidence rates of UTIs were detected. This could indicate that healthcare staff were able to keep up preventive measures against UTIs even during the pandemic challenges.

The COVID-19 pandemic, as well as non-pharmaceutical interventions and changes in personal behaviour connected to the pandemic, led to mixed effects on other infectious diseases [20,21,22]. Whereas respiratory infections decreased in a German and a Japanese study, tick-borne infections increased during the pandemic [20,21]. A Chinese study found that diarrheal disease decreased overall during the pandemic, with an increase in non-typhoidal salmonella occurring at the same time [22]. It is unclear how the pandemic influenced other infectious diseases in the context of our four LTCFs.

The annual rate of systemic prescriptions varied from 1.56/1000 resident days in 2021 to 2.13/1000 resident days in 2019. The changes over time are similar compared to the incidence rate of HCAIs with a peak in 2019 and a trough in 2021, although the decreases in incidence rates and prescription rates were not statistically significant. This highlights the close relationship between infection incidence and antimicrobial use.

Beta-lactams were the most frequently prescribed antimicrobial substances in our study. This is line with data from the most recent Austrian national report on antimicrobial resistance and antimicrobial use from 2021 [23]. This report documents antimicrobial use in all settings (in-patient and out-patient). As in the years before, beta-lactams were the most frequently used antimicrobial substance group in Austria from 2019 to 2021 [23,24]. A European point prevalence study on antimicrobial use in LTCFs described similar findings for Austria, as well as on a European level [25].

A significant decrease in the prescription rates of quinolones was noted between 2018 and 2022. In 2019, the European Commission decided on restrictions on the use of oral and intravenous quinolones stating that quinolones should be used with special caution in the elderly [26]. This recommendation could have impacted prescription rates. In addition, an antimicrobial stewardship project was conducted at the four LTCFs from January 2021 to June 2022 [27]. The project aimed at increasing the number of appropriate antimicrobial treatment courses prescribed for UTIs in LTCFs and at decreasing the proportion of quinolones used for UTIs without an indwelling urinary catheter by using a multi-faceted antimicrobial stewardship (ASP) intervention. These interventions could have contributed further to the decrease in quinolone use. On the contrary, a statistically significant increase in the rate of beta-lactam prescriptions/1000 resident days occurred between 2018 and 2022. This increase could also be associated with the ASP project, as pivmecillinam was one of the recommended first-line treatments for uncomplicated UTIs [27].

This study has some limitations. First, our findings may not be generalisable because only four LTCFs, all operated by the Geriatric Health Centres in Graz participated in this surveillance project. Second, local nursing staff recorded HCAIs only when the patient’s general practitioner diagnosed an infection. Therefore, infections, such as upper respiratory tract infections or mild skin infections, that did not need medical attention were not recorded, which could have led to an underestimation of RTIs and HCAIs in general. Third, we excluded data from LTCF 4 from July–December 2018 due to a catheter-care intervention. This may have distorted baseline incidence. However, we expect that by using incidence rates per 1000 resident days as outcome measure this possible bias would be small. Lastly, the COVID-19 pandemic significantly impacted our results. It is therefore not possible to assess whether the implementation of surveillance led to an improvement in infection control and prevention and a reduction in HCAIs.

## 4. Materials and Methods

### 4.1. Setting

The Geriatric Health Centres in Graz are a local facility comprising, among others, four LTCFs (total of 400 beds). Each LCTF is structured into smaller units of 13 to 15 residents who share a common living room.

The LTCFs assist the residents with activities of daily living and provide skilled nursing care (e.g., use of urinary catheters, wound treatment, enteral feeding tubes) as required. On-site medical care is not delivered, as the facilities do not have a centralised medical service. Instead, residents may keep their preferred general practitioners after moving to the LTCF. This means that seven to fifteen different general practitioners per LTCF provide medical care at the institution during scheduled and, if needed, on demand visits. Table 3 summarizes the characteristics of the four LTCFs. Members of the infection control and prevention (IPC) team are regularly on-site to support local staff and to review ICP practices. Annual on-site training on infection control measures for all nursing staff take place, led by members of the ICP team.

### 4.2. Data Collection and Definitions

Local nursing staff collected data on HCAIs once a week using an electronic reporting system. Collected variables included the following: age, gender, type of infection, causative agent if known, type and dose of antimicrobial therapy, as well as length of therapy. Definitions of HCAIs were based on the ECDC HALT project [28], as has been described before [11]. An HCAI was recorded only when the patient’s general practitioner was contacted by local nursing staff and diagnosed the infection, as has been described before [11]. The following entities were documented: lower respiratory tract infections (RTIs), urinary tract infections (UTIs), skin and mucosal infections (including infections of the eyes, ears and teeth), gastrointestinal infections (GTIs) and unexplained febrile illness. An HCAI was defined as a new infection if ≥7 days had passed between the end of the previous episode and the start of a new episode, as has been described before [11].

In LTCF 4, an increased rate of UTIs was detected in the first half of 2018. Therefore, the local infection control team started an intervention, including a review of existing guidelines, especially on the handling and care of indwelling catheters, as well as audits and on-site training. We therefore chose to exclude the data from LTCF 4 from July to December 2018 from our analysis.

Antimicrobial substances were categorised into five groups: beta-lactams, quinolones, folate antagonists, tetracyclines and other substances. This last category included macrolides, lincosamids, nitrofurantoin, fosfomycin, glycopeptides, rifamycin and oseltamivir. Only prescriptions for systemic anti-infective agents are included in this study.

### 4.3. Statistical Analysis

Log-linear Poisson models for counts were applied mostly to evaluate the difference effects of the various calendar years compared to the reference year of 2018. As data was available for each quarter of a year, we have also used this finer time resolution and considered generalised additive models that behave smoothly over these 20 time points [29]. Reported *p*-values refer to two-sided alternatives and are considered significant if *p* < 0.05. All the statistical analysis was done within R 4.3.2, which is a free software environment for statistical computing and graphics [30].

## 5. Conclusions

The incidence rate of HCAIs varied over time, with a significant increase during the COVID-19 pandemic in 2022 compared to 2018. UTIs were the most commonly recorded infections. No changes in the incidence rates of UTIs occurred during the study period from 2018 to 2022. The rate of beta-lactam prescriptions increased whereas the rate of quinolone prescriptions decreased. Continued surveillance efforts are necessary to assess the effect of infection control efforts after the pandemic and to monitor antimicrobial use further.

## Figures and Tables

**Table 1:** Overall incidence rate of healthcare-associated infections per 1000 resident days from 2018 to 2022 with 95% confidence intervals.

2018	2019	2020	2021	2022
2.10 (1.86, 2.38)	2.86 * (2.59, 3.16)	2.44 (2.19, 2.71)	1.86 (1.64, 2.11)	4.09 * (3.71, 4.48)

* *p* < 0.001 compared to 2018.

**Table 2:** Rate of systemic antimicrobial prescriptions per 1000 resident days from 2018 to 2022 with 95% confidence intervals.

2018	2019	2020	2021	2022
1.77 (1.54, 2.02)	2.13 * (1.9, 2.39)	1.81 (1.6, 2.05)	1.56 (1.36, 1.79)	1.88 (1.64, 2.15)

* *p* < 0.05 compared to 2018.

**Table 3:** Demographics of long-term care facilities.

	Number of Beds	Number of GPs *	Resident Days (Total 2018–2022)	Occupancy Rate in % (Average 2018–2022)	Admissions/Year (Average 2018–2022)
LTCF 1	97	11	163.322	90.2	31.6
LTCF 2	90	15	151.970	93.7	29.8
LTCF 3	104	7	181.350	96.9	36.4
LTCF 4	97	11	141.115	88.1	29.6

GP = general practitioner; * Number of GPs active in each LTFC as of 28 January 2021.

## Data Availability

The raw data supporting the conclusions of this article will be made available by the authors on request.
